# Deep Penetrating and Sensitive Targeted Magnetic Particle Imaging and Photothermal Therapy of Early‐Stage Glioblastoma Based on a Biomimetic Nanoplatform

**DOI:** 10.1002/advs.202300854

**Published:** 2023-05-07

**Authors:** Xiazi Huang, Hui Hui, Wenting Shang, Pengli Gao, Yingying Zhou, Weiran Pang, Chi Man Woo, Jie Tian, Puxiang Lai

**Affiliations:** ^1^ Department of Biomedical Engineering The Hong Kong Polytechnic University Hong Kong 000000 China; ^2^ CAS Key Laboratory of Molecular Imaging Institute of Automation Chinese Academy of Sciences Beijing 100190 China; ^3^ Hong Kong Polytechnic University Shenzhen Research Institute Shenzhen 518000 China; ^4^ Photonic Research Institute Hong Kong Polytechnic University Hong Kong 000000 China

**Keywords:** biomimetic nanoplatform, brain–blood‐barrier breaking, cancer diagnosis, glioblastoma multiforme, magnetic particle imaging

## Abstract

Early diagnosis can effectively improve the survival of glioblastoma multiforme (GBM). A specific imaging technique that is simultaneously deep penetrating and sensitive to small tissue changes is desired to identify GBM. Due to its excellent features in signal contrast, detection sensitivity, and none or little attenuation in tissue, magnetic particle imaging (MPI) possesses great potential in cancer diagnosis, especially when the imaging modality is equipped with specifically targeted nanoprobes. However, when gliomas are small, the blood–brain barrier (BBB) is complete and prevents nanoprobes from entering the brain, which negates the theranostic effect. This study proposes a biomimetic nanoplatform that assist the MPI tracers in breaking through the BBB and then demonstrate a targeted and sensitive diagnosis of GBM. Afterward, the photothermal therapy and immune regulation show an excellent therapeutic effect on the GBM. It is experimentally confirmed that the MPI signal does not decay with tissue depth and shows excellent sensitivity for thousands‐cells. Only small animals are conducted in this study due to the limitations of the current commercial MPI scanner, however, this research theoretically enables large animal and human studies, which encourages a promising pathway toward the noninvasive diagnosis of early‐stage GBM in clinics.

## Graphical abstract

Magnetic particle imaging possesses supreme sensitivity to respond to as few as thousands‐cells in vivo, and such a capability does not decay with increased tissue depth. It shows promising potential to diagnose early‐stage glioma based on homogenous‐targeting cell membrane‐functionalized nanoplatforms driven by the brain–blood‐barrier breaking. The proposed platform sees no theoretical restriction for large animal and human studies.

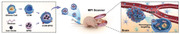

## 1 Introduction

Glioblastoma multiforme (GBM), a grade IV astrocytoma in the World Health Organization (WHO) Classification of Tumors of the Central Nervous System, is one of the most aggressive malignancies;^[^ ^1^ ^]^ the average 5‐year survival rate for patients with glioblastoma is only 6.8% because the diffusion may blur the boundary between the tumor area and normal tissue, resulting in an ineffective surgical excision. That said, if it is diagnosed early and treated appropriately, the survival period of gliomas can be considerably prolonged.^[^ ^2^ ^]^ Therefore, it is imperative to establish early, stable, and reliable cancer screening methods to prolong the survival of GBM patients.^[^ ^3^ ^]^ However, conventional imaging methods, such as magnetic resonance imaging (MRI) and X‐ray computed tomography (CT), are inaccurate in determining the size and location of brain tumors, while optical imaging is constrained by strong scattering of light in tissue and hence limited tissue penetration depth, even at near‐infrared (NIR) II wavelengths.^[^ ^4^ ^]^ More importantly, these techniques lack the required sensitivity and specificity for predicting tumor aggressiveness and differentiating tumor progression from nonspecific treatment‐related adjustments.^[^ ^5^ ^]^ According to the Gompertzian growth curve for solid tumors, the proliferation of tumor cells triggers angiogenesis when the number of cells is around 1 × 10^5^, which in turn promotes the formation of the tumor microenvironment, logarithmic increase of tumor cells, and eventually the tumor growth.^[^ ^6^ ^]^ For early‐stage tumors that are geometrically smaller than 1 mm^3^, they can hardly be detected by present imaging techniques.

Magnetic particle imaging (MPI) is an emerging tracer‐based noninvasive imaging technology that was first introduced in 2005.^[^ ^7^ ^]^ MPI has the unique advantage of no background tissue signal and excellent imaging contrast due to the limited existence of superparamagnetic nanoparticles in native biological tissues, which is different from the universal distribution of ^1^H in water and biological tissues as detected in MRI.^[^ ^8^ ^]^ So far, MPI has been exploited for different applications such as cardiovascular, cerebral ischemia, and pulmonary imaging.^[^ ^9^ ^]^ More promisingly, MPI overcomes the penetration depth limitation of optical imaging and exhibits high sensitivity, being able to quantize as few as 200 cells,^[^ ^10^ ^]^ which has immense potential for early‐stage cancer diagnosis.^[^ ^11^ ^]^

MPI signals are directly generated from the superparamagnetic nanoparticles that nonlinearly respond to magnetic field changes.^[^ ^12^ ^]^ Superparamagnetic iron oxide (SPIO) nanoparticle is a common MPI tracer that is non‐radioactive, non‐degradable over time, biocompatible, and has obtained FDA approval for clinical usage.^[^ ^13^ ^]^ However, thus far there has been no specific targeting ligand to functionalize MPI nanoprobes for in vivo glioma applications. To improve the performance of MPI for precise diagnosis of brain tumors, biomimetic materials become the prior choice due to their intrinsic immense advantages.^[^ ^14^ ^]^ For example, cell membranes derived from the organism have special biological functions such as long‐term circulation and immune regulation.^[^ ^15^ ^]^ Biomimetic membrane‐functionalized nanoprobes are born with outstanding biocompatibility and less clearance to the immune system.^[^ ^16^ ^]^ Moreover, conventional nanomaterials cannot effectively penetrate the tumor through the enhanced permeability and retention (EPR) effect alone due to the heterogeneity and unique blood–brain barrier (BBB),^[^ ^17^ ^]^ the primary barrier in brain applications.^[^ ^18^ ^]^ For membrane‐functionalized nanoprobes, the cell membranes characterized by the fluidity of the lipids bilayer and transmembrane proteins enable non‐selective nanoparticles to cross the BBB. In particular, surface antigens expressed by cancer cell membranes (CCM) have domains that bind to homologous cells, which further enhances the interaction and targeting ability with the source cells, and endows nanoparticles coated with CCM with tumor‐targeting properties.^[^ ^19^ ^]^

**Scheme 1:**
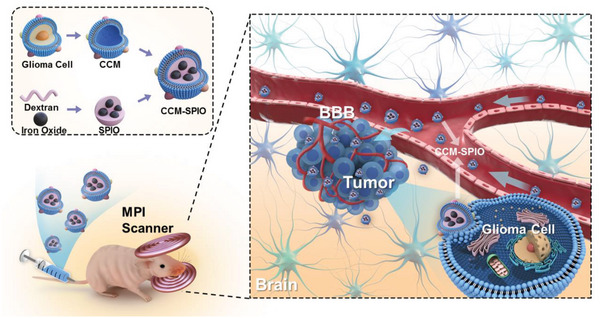
Schematic illustration of the synthetic and functional process of CCM‐SPIO nanoprobes.

In this work, we extract the membrane of glioblastoma cells and cover it on the surface of SPIO by extrusion to prepare a nanoprobe (CCM‐SPIO) that can break through the BBB and subsequently target brain glioma. This nanoprobe possesses superior magnetic and photothermal effects and can be used for early and precise detection and intervention of GBM (**Scheme** **1**). The size of CCM‐SPIO is designed to be around 90 nm, which is considered most conducive to entering the glioma.^[^ ^20^ ^]^ In vivo and in vitro tests are designed to show that the probe can pass the BBB and has the ability to target glioblastoma. MPI and fluorescence imaging (FI) are used to accurately image mice infected with glioma and to study the in vivo behavior of the nanoprobe. In addition, the enhanced immune response provided by the CCM and the preeminent photothermal effect provided by the SPIO core makes the nanoprobe a promising therapeutic agent. Furtherly, satisfactory photothermal therapy (PTT) effect on mouse glioma models is demonstrated and elevated immune response in the therapeutic groups is revealed. It is collectively implied that the emerging MPI technique equipped with the proposed biomimetic BBB‐breaking nanoplatform can provide new insights and pathways to the noninvasive diagnosis and targeted treatment of in situ early‐stage GBM.

## 2 Results and Discussion

### 2.1 Characterization of CCM‐SPIO

**Figure 1:**
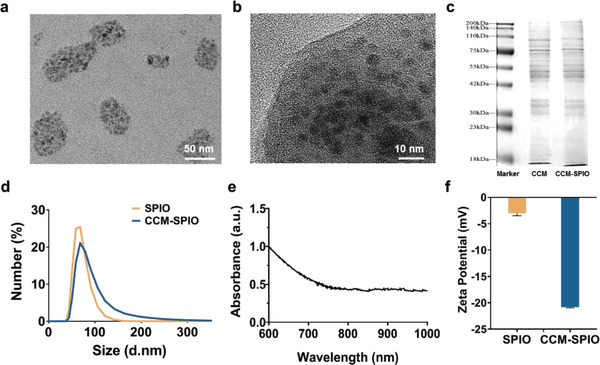
Characterization of the CCM‐SPIO nanoprobes. a,b) TEM images of CCM‐SPIO. c) SDS‐PAGE protein analysis results of cancer cell membrane vesicles and CCM‐SPIO. d) DLS results of CCM‐SPIO nanoprobes. e) UV–vis‐NIR spectra of CCM‐SPIO suspensions. f) Zeta potential of SPIO and CCM‐SPIO nanoprobes (*n* = 3).

We tested the morphology, protein separation, hydrodynamic size, surface zeta potential, and absorption spectrum using TEM, SDS‐PAGE, DLS, and UV–vis‐NIR, respectively, to characterize the synthesized nanoprobe. TEM observations (**Figure** **1**a,b) display that CCM‐SPIO represents a distinctive core–shell, and a translucent membrane structure was added compared with that of SPIO (Figure S1, Supporting Information). The unique biometric characteristics of CCM‐SPIO are verified by SDS‐PAGE. The total protein extracted from CCM‐SPIO was identical to the complete protein in the original GL261 cell membrane (Figure 1c), suggesting that the biomimetic nanoprobe ultimately inherits the protein characteristics of the original cell and provides the basis for the in vivo targeting effect. The hydrodynamic size of CCM‐SPIO was ≈87.4 nm, which increased by 18.2 nm to the SPIO core (69.2 nm) (Figure 1d). The result consists of the thickness of cell lipid bilayer membranes, which is well‐known to be 5–10 nm thick.^[^ ^21^ ^]^ Small nanoparticles of this size facilitate the EPR effect to penetrate the blood–brain tumor barrier to enhance passive aggregation in GBM tumors.^[^ ^17^, ^22^ ^]^ Additionally, it has been reported that nanoparticles larger than 100 nm in size can be occluded because glioblastoma cells have characteristic pore cutoff sizes ranging from 7 to 100 nm.^[^ ^20^ ^]^ The optical absorption spectrum of CCM‐SPIO is exhibited in Figure 1e, which presents a smooth absorption from 700 to 1000 nm, making it suitable for NIR optical therapy at depths. The stability of CCM‐SPIO was tested by monitoring the change in optical absorption spectra of the solution with PBS/FBS/DMEM. From Figure S2, Supporting Information, we can see that the absorption spectra of the three solutions mentioned above were almost identical from different measurements, attesting to a stable structure of CCM‐SPIO in PBS/FBS/DMEM. The outcome implies that CCM‐SPIO can maintain effectiveness and performance in vivo. The zeta potential of the CCM‐SPIO changed from −3 to −20.8 mV after camouflaging (Figure 1f); the latter was similar to the surface charge of the cell membrane.^[^ ^23^ ^]^ Such a change in zeta potential makes the fabricated magnetic particles more biocompatible and more suitable for in vivo applications.^[^ ^24^ ^]^ Characterization analysis showed that CCM‐SPIO was successfully synthesized and retained the protein characteristics of the cell membranes.

### 2.2 BBB Breaking and Homogenous Targeting of CCM‐SPIO

**Figure 2:**
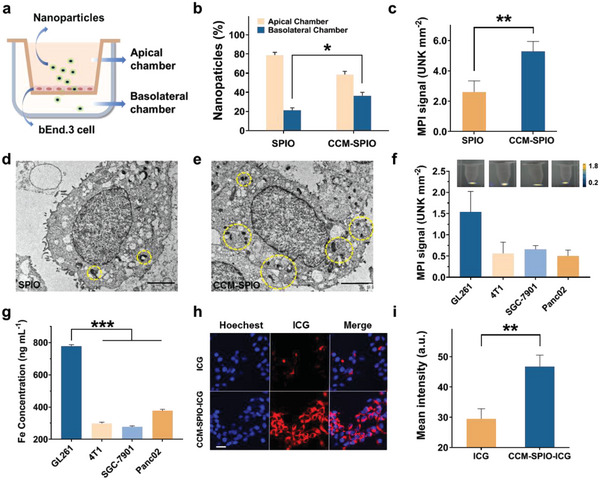
a) Schematic of the in vitro transwell model. b) Transcytosis efficiency of various formulations in the BBB model measured by ICP. c) MPI quantitative analysis of glioma cells treated with SPIO and CCM‐SPIO. d,e) TEM images of glioma cells treated with SPIO and CCM‐SPIO. f) MPI images and quantitative analysis of four different tumor cells after co‐incubation with CCM‐SPIO. g) Quantitative Fe analysis of cells in (f) by ICP. h) CLSM images of glioma cells treated with ICG and CCM‐SPIO‐ICG nanoprobes, respectively (the blue indicates nucleus stained with Hoechst, the red indicates ICG). Scale bar = 20 µm. i) Quantitative analysis of CLSM images in (h).

To evaluate whether the CCM coating could enhance the nanoprobe to break through the BBB, which is a major biological barrier that prevents nanoparticles from targeting and accumulating in gliomas, we simulated an in vitro BBB model using bEnd.3 cells as illustrated in **Figure** **2**a.^[^ ^25^ ^]^ The results showed that more nanoprobes could cross the tightly connected vascular endothelial cells after being coated with CCM (36.5% ± 2.5 vs 21% ± 2.0 at 8 h, *p = 0.0401, Figure 2b), indicating that CCM‐SPIO can better penetrate the BBB, and improve the utilization in early‐stage gliomas with intact BBB. In addition to the BBB penetration, modification of CCM is expected to render a nanocarrier with tumor‐targeted ability since the homogenous aggregation property of cancer cells. To demonstrate this, we compared the amount of SPIO and CCM‐SPIO taken up by glioma cells in vitro. As seen (Figure 2c), the MPI signal of glioma cells co‐cultured with CCM‐SPIO is much higher than that of SPIO (5.286 ± 0.665 vs 2.623 ± 0.722, **p = 0.0093), suggesting that CCM cloaking promotes the accumulation of nanoprobes in the tumor. In addition, biological electron microscopy also intuitively showed that many more CCM‐SPIO were phagocytized by the glioma cells than unmodified SPIO (Figure 2d,e), further confirming improved tumor targeting by CCM cloaking.

Subsequently, four different kinds of tumor cells (4T1, GL261, Panc02, and SGC‐7901) were incubated with CCM‐SPIO separately to verify that nanoparticles derived from tumor cell membrane modifications can promote the uptake by homologous cells and improve targeting properties. It should be specified here the membrane source of CCM‐SPIO was extracted from GL261 cells, so the synthesized nanoprobe is expected to target the GL261 cells. Detailed experimental procedures are presented in the Experimental Section. After removing the supernatant, the cells were digested, collected, and tested for MPI signal, respectively. It can be seen from the results that the signal of magnetic nanoparticles in glioma cells is the highest (Figure 2f). Consistent results were obtained by ICPICP quantification of Fe content in the cell precipitates (Figure 2g). The Fe content in glioma cells was significantly different from that in the other three groups (***p < 0.001). These indicate that the modified SPIO has a considerably improved targeting ability to target protein‐derived glioma cells but has no binding ability to other types of tumor cells, that is, outstanding specificity to glioma cells. We also studied the fortified targeting ability of ICG‐marked CCM‐SPIO with CLSM. The absorbance of the ICG‐marked CCM‐SPIO is endowed with the characteristic absorption peak of ICG, indicating that the ICG was successfully loaded into the membrane layer (Figure S3, Supporting Information). As expected, CLSM images showed stronger signals from the ICG channel in the ICG‐marked CCM‐SPIO group, indicating that the nanoprobes were effectively internalized into the glioma cells (Figure 2h). The mean fluorescent intensity between the two groups was statistically significant, again illustrating that many more nanoprobes were attached to the tumor cells after being wrapped with CCM (**p = 0.0041, Figure 2i). These experiments revealed the feasibility of a tumor cell membrane encapsulation scheme to improve the glioma‐specific targeting of nanoparticles.

### 2.3 Sensitivity and Deep Penetration Demonstration of MPI

**Figure 3:**
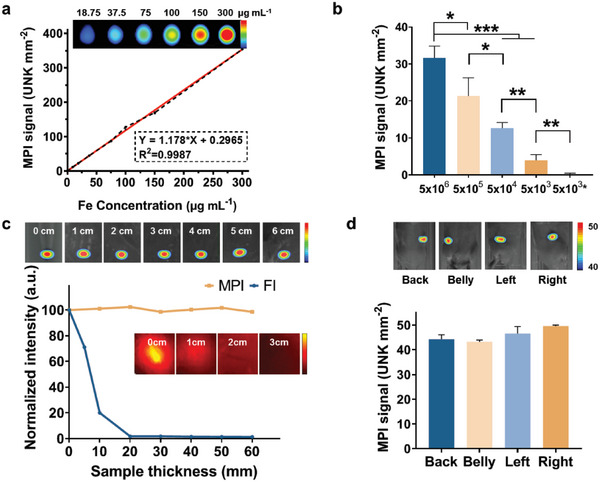
MPI sensitivity and penetration testing with CCM‐SPIO. a) In vitro MPI images of CCM‐SPIO at different Iron concentrations (0, 25, 50, 100, 150, and 200 µg mL^−1^). b) MPI signals of a variety amount of GL261 cells post co‐incubate with CCM‐SPIO. The last group 5 × 10^3^* means cells without nanoprobe. c) Intensity changes of MPI and FI with gradually increased thickness of chicken breast overlaid on the nanoprobes. d) MPI images of mouse subcutaneous glioma models at different positions. The error bars represent the standard deviation from measurements with *n* = 3.

As an emerging imaging technique, MPI is considered to have exceptional sensitivity and penetrating properties in tissue. Hence, we first tested the MPI performance of CCM‐SPIO in vitro to verify MPI's detection limit. As shown in **Figure** **3**a, the MPI signal of the nanoprobes increased approximately linearly with the iron concentration in the probe. With such a linear relationship, the nanoprobes in the region of interest (ROI) can be quantitatively analyzed according to the MPI signal intensity. Pellets with increasing numbers of CCM‐SPIO‐loaded glioma cells were examined to determine the minimum number of cells that could be detected by MPI (Figure 3b) in this study. Overall, the MPI signal decreased significantly with reduced cell number, indicating that MPI could further distinguish different numbers of tumor cells labeled with CCM‐SPIO. Moreover, it can be seen that the MPI signal of 5 × 10^3^ tumor cells co‐incubated with CCM‐SPIO was significantly stronger than that of an equal number of uninfected cells (5.333 ± 1.528 vs 0.133 ± 0.351, **p = 0.0045). The pellet containing 5 × 10^4^ tumor cells labeled with CCM‐SPIO was clearly distinguished from that containing 5 × 10^3^ cells (12.667 ± 1.528 vs 5.333 ± 1.528, **p = 0.0042). Due to the inevitable background noise from the MPI scanner and the difference of the probe phagocytosis in cells, the smallest amount of CCM‐SPIO‐labeled tumor cells that can be detected by MPI in our study is 5 × 10^3^, which is one‐quarter of the detection limit of 2 × 10^4^ cells reported in the literature for MRI.^[^ ^26^ ^]^ This proves the supreme sensitivity of MPI assisted by CCM‐SPIO, which is essential to assure the detection of early‐stage primary tumors.

The other important feature to enable the diagnosis of early‐stage glioblastoma in vivo is the tissue penetration capability of the imaging modality. To demonstrate that of MPI, we chose FI, one of the most popularly used imaging modalities in the field, as the reference. As known, FI can sensitively detect signals from biological tissues, which, however, is limited to the shallow depth of a few millimeters beneath the tissue surface and hence encounters insufficiency in large animals or humans. In Figure 3c, we compared the influence of tissue sample thickness to in vivo MPI (the top panel) and FI (the inset panel, with ICG loaded onto the CCM‐SPIO probes) on glioma‐bearing mice, by examining the changes of signal from samples covered by chicken breast tissue of varying thickness (0–6 cm). As expected, the MPI signal remained nearly constant with increased sample thickness, whereas the intensity of FI dropped rapidly to ≈20% when the sample thickness was 1 cm and almost to zero when the thickness was increased to 2 cm. Additionally, the mouse tumor model labeled with CCM‐SPIO was investigated by placing the animal model at several positions consisting of different tissue depths for the tumor site. As seen (Figure 3d), there are no significant variations among the MPI signals with different tissue depths. These results in combination demonstrate the immunity of MPI to sample thickness and positioning, which is equally if not more than, important to assure MPI for deep tissue applications.

### 2.4 Multimodal Imaging of Glioma Allografts in Mice

**Figure 4:**
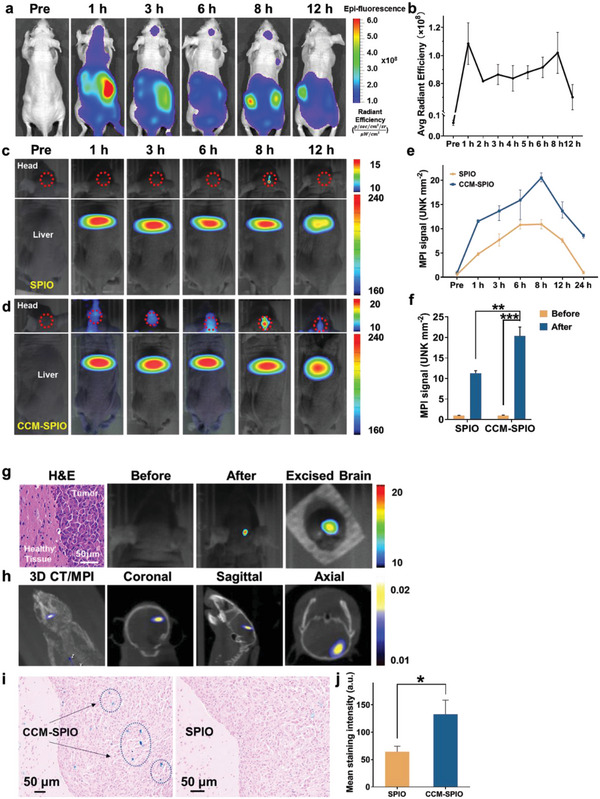
Multimodal imaging of orthotopic brain tumor xenografts in Mice. a) Fluorescence images of GL261 tumor‐bearing mice at different time points (pre‐injection, 1, 3, 6, 8, and 12 h), and b) the corresponding fluorescence intensities at the brain tumor site (*n* = 3). 2D MPI images of a mouse head with an orthotopic brain tumor after injection of SPIO (c) and CCM‐SPIO (d), respectively. The dashed red circles represent the ROI. e) The corresponding quantified analysis of brain MPI signal at different time points. f) Brain MPI signal intensities before and 8 h post the injection of SPIO and CCM‐SPIO, respectively. g) H&E staining and 2D MPI images of the brain from the mice injected with CCM‐SPIO. h) 3D MPI/CT images of mice head with glioma before and 8 h post the injection of CCM‐SPIO. i) Prussian blue staining of tumor sections at 8 h post the injection of CCM‐SPIO. j) The corresponding quantification of Prussian blue staining signals from the brain regions.

To further evaluate the deep‐tissue tumor imaging capability of CCM‐SPIO, a mouse orthotopic glioblastoma tumor model was built (Figure S4, Supporting Information) and injected with CCM‐SPIO. Initially, the fluorescent images demonstrated the bio‐distribution of ICG‐marked CCM‐SPIO, which rapidly distributed throughout the body with the blood flow after intravenous injection and was subsequently excreted (**Figure** **4**a,b and Figure S5, Supporting Information). It was observed to be fully distributed in the brain within an hour, and the signals of tumor areas gradually increased over time from 1 to 8 h post‐administration, indicating that the CCM‐SPIO was efficiently delivered into the tumor. The ex vivo brain at 8 h confirmed that the ICG‐marked CCM‐SPIO was efficiently delivered into the tumor (Figure S5a, Supporting Information). Because of the relatively shallow penetration depth required in small animals, the nanoprobe can also be detected by FI and be observed to reach the brain glioma sites. The average radiant efficiency of ROI also displayed that the signal rapidly reached a maximum value at 1 h, then dramatically declined, and gradually reached another peak at 8 h, indicating that the nanoprobes accumulated at the tumor site by targeting effect (Figure 4b). Correspondingly, dynamic MPI visualized the gradual accumulation of CCM‐SPIO in tumor areas from 1 to 8 h after the intravenous injection, as illustrated in the 2D MPI images in Figure 4d. As a comparison, a relatively weak MPI signal was observed in the tumor sites at the captioned time after the injection of SPIO (Figure 4c). At the 24‐h, the signal from SPIO in the brain almost disappeared, while the signal from CCM‐SPIO remained (Figure 4e), suggesting that CCM enhanced the ability of the nanoprobe to penetrate the BBB and the target glioma. It is also possible that the increased exposure of polyethylene glycol (PEG)‐SPIO to the reticuloendothelial system could accelerate its clearance; by contrast, biofilm modification could more wittily evade the clearance by the immune system and prolong the circulation time in the body.

Quantification analysis showed a 20.6‐fold increase in MPI signals from the marked tumor regions after CCM‐SPIO injection (20.341 ± 2.187 vs 0.986 ± 0.043 at 8 h post the injection, ****p < 0.0001), which is a 1.8‐fold increase compared to that with SPIO injection (20.341 ± 2.187 vs 11.256 ± 0.662 at 8 h post the injection, **p = 0.0023, Figure 4f). It suggests that after coating, the nanoprobes could reach the mice's brain faster, accumulate more at the tumor site, and remain longer in the brain. This conclusion was also confirmed by ex vivo MPI and histopathological analysis as shown in Figure 4g. 3D CT/MPI imaging of the mouse brain was also performed (Figure 4h). With the structural information provided by CT, the location and size of the brain tumor can be clearly determined by the MPI signals from different orientations. The mice were dissected to isolate the brain after imaging. The tumors in the brain were visualized with an average diameter of 2 mm, and the maximum cross‐section of the tumor was 2 × 2.1 mm^2^ in the H&E section of the brain (Figure S6, Supporting Information).

After all images were obtained, the tumor tissues from mice were dissected and analyzed by Prussian blue staining. The results (Figure 4i) showed that the blue color was more pronounced in the tumor site in the CCM‐SPIO group than in the SPIO group, which could be further verified by the quantitative analysis (Figure 4j). These results indicate that CCM‐SPIO is more effective than SPIO for glioma targeting.

### 2.5 Toxicity, Photothermal Effect, and Cell Viability Test on CCM‐SPIO

**Figure 5:**
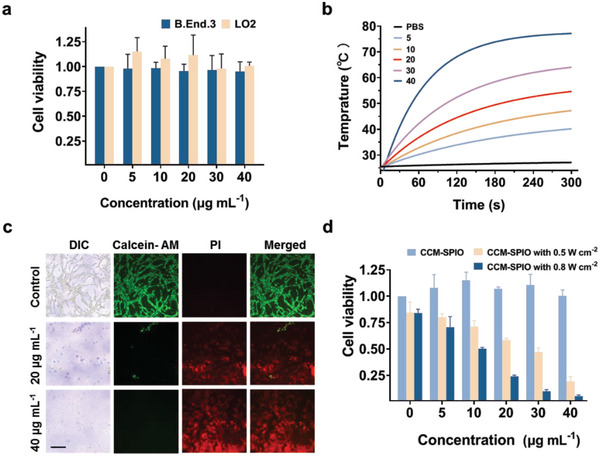
a) Toxicity of various concentrations of CCM‐SPIO on normal cells. b) The photothermal temperature‐time curves of CCM‐SPIO at different concentrations (0, 5, 10, 20, 30, and 40 µg mL^−1^) under 780 nm laser (0.8 W cm^−2^, 5 min) irradiation. c) CLSM images of cancer cells co‐stained with CAM and PI after various treatments to distinguish the live (green fluorescence) and dead (red fluorescence) cells. d) CCK8 results after various treatments (*n* = 3).

The toxicity of the synthesized nanoprobes was evaluated on the B. End.3 cells, the primary component cell of the mouse BBB, and LO2, a type of normal liver cell. As shown in **Figure** **5**a, the viability of both cell lines changed slightly after being incubated with gradually increased concentrations of the nanoprobe (0, 5, 10, 20, 30, and 40 µg mL^−1^ of iron), indicating good biocompatibility and minor toxicity of the probe. The degradation process of CCM‐SPIO in cells was observed by biological electron microscopy. It can be observed that the presence of dense aggregates of the CCM‐SPIO inside the early and late endosomes and subsequently in the lysosomes. This is consistent with the intracellular metabolism characteristics of degradable nanomaterials which are internalized primarily through the endocytic process of vesicle formation.^[^ ^27^ ^]^ Once inside the lysosome, the probe begins to degrade and disintegrate, as indicated by Figure S7, Supporting Information.

To evaluate the photothermal effect of the CCM‐SPIO, a laser beam was directed to the neck of the EP tube containing PBS/CCM‐SPIO with varying concentrations (5, 10, 20, 30, and 40 µg mL^−1^ of iron) and the temperature of the nanoprobe solution was recorded every 30 s (Figure S8, Supporting Information). As seen (Figure 5b), the temperature of the CCM‐SPIO solution exhibited a sharp increase with the exposure time, implying that it has an excellent PTT ability. The rate and final temperature of the solution increase with the concentration of CCM‐SPIO; with 10 µg mL^−1^ of iron concentration, exposure for 180 s can elevate the temperature close to 43 °C, which is sufficient to result cell apoptosis (42.75 ± 0.55 °C).^[^ ^28^ ^]^

Subsequently, the photothermal capacity of the CCM‐SPIO on the tumor cells was tested by calcein‐AM and propidium iodide (PI) staining (Figure 5c), which is responsible for visualizing live and dead cells, respectively. As seen, the cells incubated with the CCM‐SPIO were dead after 5 min of irradiation, while the control group showed superior viability. The PI fluorescence in the group with higher concentration (40 µg mL^−1^) is much stronger than that in the lower concentration (20 µg mL^−1^) group, which agrees with the tendency of Figure 5b. The cell viability after irradiation was also evaluated by CCK‐8 assay (Figure 5d), where the cell viability was significantly reduced after co‐incubation with CCM‐SPIO and irradiation, further demonstrating PTT's lethal effect on cells. Cell viability decreased with the increased iron concentration and irradiation intensity (0.1935 ± 0.044 at 0.5 W cm^−2^ and 0.0522 ± 0.012 at 0.8 W cm^−2^ with 40 µg mL^−1^ concentration). These results indicate that CCM‐SPIO has a minimal cytotoxic effect on living cells without irradiation, but it could effectively eliminate tumor cells under light irradiation of 0.8 W cm^−2^ for 5 min, showing promising PTT competence.

### 2.6 Tumor Treatment Effect In Vivo

**Figure 6:**
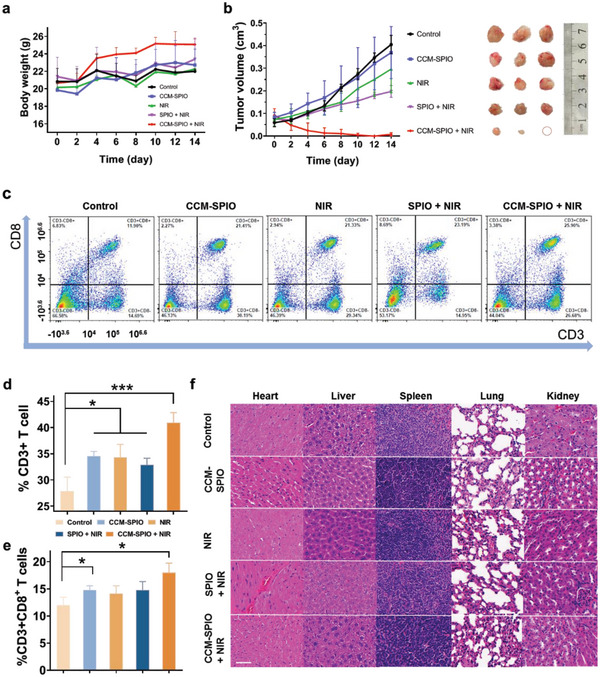
Anti‐tumor effects of PTT based on CCM‐SPIO on glioma mouse models. a) Change of mice weight during treatment. Data are expressed as the mean ± SD (*n* = 4). b) Changes in tumor volumes over time and representative photographs of tumors in each group. c) Representative populations of CD3+CD8+ CTLs isolated from LNs at 14 days post‐treatment in each group. d,e) Percentage of CD3+and CD3+CD8+CTLs in spleens and LNs in each group. f) Representative H&E staining images of the tissues of each group. The scale bar = 100 µm.

We further studied the antitumor treatment efficacy of CCM‐SPIO on the glioblastoma subcutaneous xenograft tumor model in C57/6N mice. The mouse body weight variation was recorded every other day for 2 weeks from the day of treatment. During monitoring, all mice behaved normally and had no significant weight loss (**Figure** **6**a), indicating negligible toxicity of the CCM‐SPIO. The mice in the treatment group gained a slight amount of weight, likely as a result of their improved health condition after tumor suppression. As shown in Figure 6b, after 785 nm irradiation (0.8 W cm^−2^), the tumor volumes in the treatment group (CCM‐SPIO + NIR) were significantly suppressed, while the tumors in other groups showed malignant growth, demonstrating that the superior therapeutic effect of CCM‐SPIO. Tumor shrinkage in the treatment group (CCM‐SPIO + NIR) reached 92 ± 0.071%, and the antitumor effect was the best. After all monitoring studies, the tumor sites were dissected (the rightmost panel in Figure 6b) to measure their weights (Figure S9, Supporting Information). The obtained average tumor weight of the treatment group (CCM‐SPIO + NIR) diminished to 0.033 ± 0.058 g, which is more than one magnitude lower than that from all other groups, such as the control group (0.900 ± 0.100 g, ***p = 0.0002) and SPIO group (0.500 ± 0.100 g, **p = 0.0022,). These results indicate that PTT with CCM‐SPIO has excellent tumor therapeutic performance.

In addition, as the CCM‐modified nanoprobes were expected to induce cancer antigen immunotherapy,^[^ ^29^ ^]^ spleens, inguinal lymph nodes (LNs), and tumors were dissected at 14 days after the therapy and digested into individual cell suspensions for flow cytometry analysis to measure the percentage of the cytotoxic T‐lymphocytes (CTL). The results (Figure 6d,e) showed that the CTL‐mediated antitumor response was elicited after treatment, as reflected by a remarkably increased percentage of CD3+CD8+T cells (*p = 0.0102). Some representative images are shown in Figure 6c. Notably, mice injected with CCM‐SPIO also displayed a significantly increased percentage of CD3+CD8+T cells compared with the control group, which could be considered as evidence of immunotoxicity induced by CCM (*p = 0.0414). After observing the indication of a systemic immune response, we further profiled CTL entering the tumors. It is found (Figure S10, Supporting Information) that a significantly increased proportion of cytotoxic CD3+CD8+T cells infiltrated the treated tumors following PTT with CCM‐SPIO compared with the other groups. A corresponding significant decrease in CD3+CD4+T cells was also analyzed in more detail, which is considered to be suppression of T‐regs responsible for inhibiting the antitumor immune responses (Figure S11, Supporting Information). Overall, the evidence of the shifts in CD4+ and CD8+T cell populations was consistent with the hypothesis that CCM‐SPIO can induce positive immunomodulation following PTT, assisting in achieving better therapeutic effects. Last but not least, the major organs of the mice were investigated by H&E staining at the end of the treatment to determine the biocompatibility and safety after the different groups of treatment. The results (Figure 6f) displayed no apparent damage to the major organs of the mice, further confirming the low systemic toxicity in vivo of the synthesized CCM‐SPIO nanoprobes.

## 3 Discussion and Conclusion

In this work, based on an enveloped bioinspired design strategy, we have developed a CCM‐coated SPIO nanoprobe for sensitive and deep‐penetrating MPI diagnosis and effective PTT of early‐stage glioma. The resultant formulation (CCM‐SPIO) exhibited favorable BBB breaking and homologous tumor‐targeting intelligence owing to the CCM coating. Compared to commercially available SPIO, the nanoprobe can more effectively cross the BBB and be encapsulated by the glioma cells, endowing noninvasive MPI with the capability to diagnose early‐stage glioma. More importantly, with the assistance of the nanoprobe, the sensitivity and deep penetrating features of MPI were demonstrated. Notably, the smallest amount of CCM‐SPIO‐labeled tumor cells that can be detected in this study is as low as 5 × 10^3^, which is 25% of the detection limit with the state‐of‐the‐art MRI and 5% of the threshold that triggers tumor angiogenesis (1 × 10^5^),^[^ ^6^, ^30^ ^]^ displaying convincing basics for the early detection of glioma. The targeting ability of the CCM‐SPIO was tested by MPI on orthotopic glioma mouse models. It was found that CCM‐SPIO reached the mice's brain faster, was more potent at the tumor site, and remained in the brain longer compared with non‐modified SPIO. The MPI signal of the brain tumor increased more than 20 times after the injection of the CCM‐SPIO, manifesting outstanding glioma‐targeting competence of the nanoprobe. In addition, benefitting from the excellent photothermal effect of the inner SPIO of the nanoprobe in the NIR absorbance band, we could have effective PPT on mouse glioma models, with results demonstrating the enhanced antitumor effect with CCM‐SPIO accumulation at the tumor site. Collectively, the strategy of combining biomimetic cell membrane coating with magnetic nanoparticles provides indispensable pathways in MPI for deep‐penetrating and sensitive tissue applications. Once patient‐specific CCM can be obtained from the source cancer cells, it may be of greatest interest to address the cancer heterogeneity arising from the coexistence of multiple cell types with different phenotypes and improve patient‐specific treatment and promote the development of personalized treatment. Finally, it should be noted that we were unable to work with large animals in this study due to the space confinement of the current MPI system, which, however, is not bounded in principle (as demonstrated in Figure 3c,d) and can be extended for large animals or even human applications once the MPI system can be engineered to hold large samples. It is collectively implied that the proposed platform can provide new insights and pathways to the noninvasive diagnosis and targeted treatment of in situ early‐stage GBM.

## 4 Experimental Section

### Synthesis and Characterization of CCM‐SPIO

CCM vesicles were extracted using the Membrane and Cytosol Protein Extraction Kit (Beyotime P0033). Briefly, the adherent cells were washed with PBS, scraped off with a cell scraper, and blown down with a pipette. The cells were collected by centrifugation (1000 rpm, 3 min), then the supernatant was removed, and the cell precipitate was left for later use. After the collected cells were washed with cold PBS, they were fully suspended in membrane protein extraction reagent A with 1% PMSF. Then, the suspended cells were transferred into the glass homogenizer for 60‐time homogenization after being pre‐cooled in an ice bath for 15 min. The supernatant was collected under centrifugation at 700 g (4 °C, 10 min), and the cell membrane fragments were precipitated under centrifugation at 14 000 g (4 °C, 30 min). The collected CCM was suspended in PBS and sonicated in a glass bottle for 30 min using a bath sonicator (Fisher Scientific, Waltham, MA, USA) at 42 kHz and a power of 100 W under 4 °C. The resultant CCM vesicles were extruded sequentially through polycarbonate porous membranes with a pore diameter of 1000, 600, 400, 200, and 100 nm using an Avanti mini extruder (Avanti Polar Lipids, Alabaster, AL, USA). The prepared CCM vesicles and SPIO solution (VivoTrax, Magnetic Insight, Inc., Alameda, CA, USA) were then mixed at a surface ratio of 1:1 (*S*_CCM_/*S*_SPIOs_), following the surface area analysis.^[^ ^15a^ ^]^ The mixture was sonicated for 30 min under 4 °C, then extruded sequentially through 200‐ and 100‐nm polycarbonate porous membranes. The extrusion process was repeated three times to ensure uniform and good dispersion of the synthesized nanoparticles.

### In Vitro Targeting Studies

To verify that CCM could enhance the tumor‐targeting ability of SPIO, glioma cells were co‐cultured with CCM‐SPIO and SPIO for 6 h, separately. After being washed with PBS, the cells were collected for an MPI signaling test and biological electron microscopy. To further evaluate the targeting effect of CCM‐SPIO, four different kinds of tumor cells, including breast cancer cell (4T1), glioblastoma cell (GL261), pancreatic cancer cell (Panc02), and gastric carcinoma cell (SGC‐7901), were incubated overnight and co‐incubated with CCM‐SPIO for another 6 h. In the end, the cells were collected to test the MPI signals and ICP after being washed with PBS twice. On the other hand, GL261 cells were co‐incubated with ICG/CCM‐SPIO‐ICG for 6 h for confocal imaging assessment. After that, the cells were stained with Hoechst 33 258 and imaged by confocal laser scanning microscopy (CLSM, Dragonfly 200, Andor, Shenzhen, China).

### In Vitro and In Vivo MPI

MPI was performed by an MPI scanner (MOMENTUM, Magnetic Insight, Inc., Alameda, CA, USA) with a frequency of 45 kHz and a magnetic gradient strength of 6 T m^−1^. The scan mode is set as orthotropic; Z field of view: 4 cm for measuring EP tubes, 5 cm for measuring chicken breast, 3 cm for measuring mouse head, and 12 cm for measuring the mouse's whole body. Data analysis was performed using VivoQuant software (VivoQuant 4.0, Invicro, Boston, MA, USA). For the demonstration of penetration depth, the nanoprobe was tested in the capillary settled under the chicken at varying depths (1, 2, 3, 4, 5, and 6 cm) using MPI.

### In Vivo PTT

24 glioma‐bearing mice were prepared as described earlier. When the tumor volume reached about 10 mm^3^, the mice were randomly divided into four evenly numbered groups: control group, CCM‐SPIO group, laser group, and CCM‐SPIO with laser group. In the control group, the healthy mice received no treatment. In the laser group, the tumor‐bearing mice were irradiated with a 785‐nm laser (0.8 W cm^−2^) for 5 min. In the other two groups, tumor‐bearing mice received 200 µL of CCM‐SPIO (1 mg mL^−1^) intravenously, but only the mice in the CCM‐SPIO with laser group were also irradiated with a 785‐nm laser (0.8 W cm^−2^) for 5 min at 8‐h post‐injection. The temperature elevation of tumors was recorded with an infrared thermal imaging camera (FLUKE Ti25, Everett, WA, USA) during the treatment. The body weight and tumor volume of each mouse from all groups were measured every other day. Tumor volume calculation formula: *V* = Length × (Width)^2^/2. The mice were dissected after death, with tumors and major organs, including heart, liver, spleen, lung, and kidney, being harvested for histological analysis.

### Immune Response Assay

When mice were sacrificed at 14 days after CCM‐SPIO‐guided PTT, the tumor, spleen, and inguinal lymph in the groups were collected and used for flow cytometry analysis of immune cells. To each sample tube with 100 µL of cell suspension (10^7^ cells mL^−1^), 1 µg of anti‐mouse CD16/32 (TruStain FcX) was added, mixed, and incubated for 10 min at room temperature. Fluorescence‐labeled antibodies were added to the cell suspension according to the combination (CD45 BB515/CD3 PE/CD4 APC/CD8 PE‐Cy5) and incubated for 20 min at room temperature in a dark place. 2 mL of PBS containing 1% FBS was added to each sample tube. The mixtures were centrifuged at 500 g for 5 min to remove the supernatant. Then, 0.5 mL of PBS containing 1% FBS was added, and analytical data were acquired on a Cytek NL ‐CLC3000 flow cytometer (Thermofisher, USA). All animal procedures were carried out according to the guidelines approved by the Animal Ethics Committee of the Hong Kong Polytechnic University. The approval number for animal experiments is A0043201.

## Conflict of Interest

The authors declare no conflict of interest.

## Supporting information

Supporting Information

## Data Availability

Research data are not shared.

## References

[1] a) D. N. Louis , A. Perry , P. Wesseling , D. J. Brat , I. A. Cree , D. Figarella‐Branger , C. Hawkins , H. K. Ng , S. M. Pfister , G. Reifenberger , R. Soffietti , A. von Deimling , D. W. Ellison , Neuro‐Oncology 2021, 23, 1231; 34185076 10.1093/neuonc/noab106 PMC8328013

[2] a) I. Ceravolo , G. Barchetti , F. Biraschi , C. Gerace , E. Pampana , A. Pingi , A. Stasolla , Radiol. Med. 2021, 126, 1468; 34338949 10.1007/s11547-021-01401-4

[3] a) R. Weissleder , M. J. Pittet , Nature 2008, 452, 580; 18385732 10.1038/nature06917 PMC2708079

[4] a) G. Song , X. Zheng , Y. Wang , X. Xia , S. Chu , J. Rao , ACS Nano 2019, 13, 7750; 31244043 10.1021/acsnano.9b01436

[5] a) K. J. Langen , C. Watts , Nat. Rev. Neurol. 2016, 12, 375; 27282652 10.1038/nrneurol.2016.80

[6] a) P. Waliszewski , J. Konarski , Chaos, Solitons Fractals 2003, 16, 665;

[7] B. Gleich , J. Weizenecker , Nature 2005, 435, 1214. 15988521 10.1038/nature03808

[8] a) Z. W. Tay , P. Chandrasekharan , B. D. Fellows , I. R. Arrizabalaga , E. Yu , M. Olivo , S. M. Conolly , Cancers 2021, 13, 5285; 34771448 10.3390/cancers13215285 PMC8582440

[9] a) P. Szwargulski , M. Wilmes , E. Javidi , F. Thieben , M. Graeser , M. Koch , C. Gruettner , G. Adam , C. Gerloff , T. Magnus , T. Knopp , P. Ludewig , ACS Nano 2020, 14, 13913; 32941000 10.1021/acsnano.0c06326

[10] B. Zheng , T. Vazin , P. W. Goodwill , A. Conway , A. Verma , E. U. Saritas , D. Schaffer , S. M. Conolly , Sci. Rep. 2015, 5, 14055. 26358296 10.1038/srep14055 PMC4566119

[11] a) G. Song , M. Kenney , Y. S. Chen , X. Zheng , Y. Deng , Z. Chen , S. X. Wang , S. S. Gambhir , H. Dai , J. Rao , Nat. Biomed. Eng. 2020, 4, 325; 32015409 10.1038/s41551-019-0506-0 PMC7071985

[12] a) L. M. Bauer , S. F. Situ , M. A. Griswold , A. C. Samia , J. Phys. Chem. Lett. 2015, 6, 2509; 26266727 10.1021/acs.jpclett.5b00610

[13] a) S. Liu , A. Chiu‐Lam , A. Rivera‐Rodriguez , R. DeGroff , S. Savliwala , N. Sarna , C. M. Rinaldi‐Ramos , Nanotheranostics 2021, 5, 348; 33850693 10.7150/ntno.58548 PMC8040827

[14] R. Molinaro , C. Corbo , J. O. Martinez , F. Taraballi , M. Evangelopoulos , S. Minardi , I. K. Yazdi , P. Zhao , E. De Rosa , M. B. Sherman , A. De Vita , N. E. T. Furman , X. Wang , A. Parodi , E. Tasciotti , Nat. Mater. 2016, 15, 1037. 27213956 10.1038/nmat4644 PMC5127392

[15] a) X. Huang , W. Shang , H. Deng , Y. Zhou , F. Cao , C. Fang , P. Lai , J. Tian , Appl. Mater. Today 2020, 18, 100484;

[16] P. Zhang , G. Liu , X. Chen , Nano Today 2017, 13, 7. 28435439 10.1016/j.nantod.2016.10.008 PMC5396544

[17] S. K. Golombek , J. N. May , B. Theek , L. Appold , N. Drude , F. Kiessling , T. Lammers , Adv. Drug Delivery Rev. 2018, 130, 17. 10.1016/j.addr.2018.07.007 PMC6130746 30009886

[18] a) S. D. Campbell , K. J. Regina , E. D. Kharasch , J. Biomol. Screen 2014, 19, 437; 23945876 10.1177/1087057113497981 PMC4078735

[19] X. F. Bai , Y. Chen , M. Z. Zou , C. X. Li , Y. Zhang , M. J. Li , S. X. Cheng , X. Z. Zhang , ACS Nano 2022, 16, 18555. 36341683 10.1021/acsnano.2c06871

[20] K. Wiwatchaitawee , J. C. Quarterman , S. M. Geary , A. K. Salem , AAPS PharmSciTech 2021, 22, 71. 33575970 10.1208/s12249-021-01928-9 PMC8092804

[21] J. C. Harris , M. A. Scully , E. S. Day , Cancers 2019, 11, 1836. 31766360 10.3390/cancers11121836 PMC6966582

[22] Y. Yang , Z. Yan , D. Wei , J. Zhong , L. Liu , L. Zhang , F. Wang , X. Wei , C. Xie , W. Lu , D. He , Nanotechnology 2013, 24, 405101. 24029287 10.1088/0957-4484/24/40/405101

[23] Y. Zhang , M. Yang , N. G. Portney , D. Cui , G. Budak , E. Ozbay , M. Ozkan , C. S. Ozkan , Biomed. Microdevices 2008, 10, 321. 18165903 10.1007/s10544-007-9139-2

[24] M. K. Rasmussen , J. N. Pedersen , R. Marie , Nat. Commun. 2020, 11, 2337. 32393750 10.1038/s41467-020-15889-3 PMC7214416

[25] J. Xue , Z. Zhao , L. Zhang , L. Xue , S. Shen , Y. Wen , Z. Wei , L. Wang , L. Kong , H. Sun , Q. Ping , R. Mo , C. Zhang , Nat. Nanotechnol. 2017, 12, 692. 28650441 10.1038/nnano.2017.54

[26] L. Kiru , A. Zlitni , A. M. Tousley , G. N. Dalton , W. Wu , F. Lafortune , A. Liu , K. M. Cunanan , H. Nejadnik , T. Sulchek , M. E. Moseley , R. G. Majzner , H. E. Daldrup‐Link , Proc. Natl. Acad. Sci. U. S. A. 2022, 119, e2102363119. 35101971 10.1073/pnas.2102363119 PMC8832996

[27] D. Ishwar , S. Ganesh , R. Haldavnekar , K. Venkatakrishnan , B. Tan , Mater. Today Chem. 2023, 27, 101310.

[28] X. Zhu , W. Feng , J. Chang , Y. W. Tan , J. Li , M. Chen , Y. Sun , F. Li , Nat. Commun. 2016, 7, 10437. 26842674 10.1038/ncomms10437 PMC4742858

[29] a) R. H. Fang , Y. Jiang , J. C. Fang , L. Zhang , Biomaterials 2017, 128, 69; 28292726 10.1016/j.biomaterials.2017.02.041 PMC5417338

[30] A. K. Palucka , L. M. Coussens , Cell 2016, 164, 1233. 26967289 10.1016/j.cell.2016.01.049 PMC4788788

