# Supplementary material for: Deep Penetrating and Sensitive Targeted Magnetic Particle Imaging and Photothermal Therapy of Early‐Stage Glioblastoma Based on a Biomimetic Nanoplatform
Source: Adv Sci (Weinh). 2023 May 7;10(19):2300854. doi: 10.1002/advs.202300854 (PMC10323639; doi:10.1002/advs.202300854)
Supplement: Supplementary file 1 — Supporting Information [file ADVS-10-2300854-s001.pdf]

ADVANCED SCIENCE
Open Access

Supporting Information

for *Adv. Sci.*, DOI 10.1002/advs.202300854

Deep Penetrating and Sensitive Targeted Magnetic Particle Imaging and Photothermal Therapy of Early-Stage Glioblastoma Based on a Biomimetic Nanoplatform

Xiazi Huang, Hui Hui, Wenting Shang, Pengli Gao, Yingying Zhou, Weiran Pang, Chi Man Woo, Jie Tian and Puxiang Lai**

Supporting Information

Deep penetrating and sensitive targeted magnetic particle imaging and photothermal therapy of early-stage glioblastoma based on a biomimetic nanoplatform

Xiazi Huang[a, b, c#], Hui Hui[b#], Wenting Shang[b], Pengli Gao[b], Yingying Zhou[a,c], Weiran Pang[a,c], Chi Man Woo[a,c], Puxiang Lai[a, c, d]*, and Jie Tian[b]*

a. Department of Biomedical Engineering, The Hong Kong Polytechnic University, Hong Kong SAR, China.

b. CAS Key Laboratory of Molecular Imaging, Institute of Automation, Chinese Academy of Sciences, Beijing, China.

c. Hong Kong Polytechnic University, Shenzhen Research Institute, Shenzhen, China

d. Photonic Research Institute, Hong Kong Polytechnic University, Hong Kong SAR, China

# These authors contributed equally to this work.

* Corresponding email: jie.tian@ia.ac.cn and puxiang.lai@polyu.edu.hk

Methodology

Characterization of CCM-SPIO

The resultant CCM-SPIO were then re-dispersed for further characterization by transmission electron microscopy (TEM), dynamic light scattering (DLS), zeta potential determination, and ultraviolet-visible (UV-VIS) spectroscopy. The morphology of CCM-SPIO was characterized by TEM using a FEI Tecnai F20 microscope (FEI, USA) with a 300 kV acceleration voltage. Zeta potential and size were analyzed with a ZEM 3600 Malvern Zetasizer (Malvern, UK). After that, sodium dodecyl sulfate-polyacrylamide gel electrophoresis (SDS-PAGE) was utilized to characterize membrane proteins. Optical absorption spectra were measured using a UV-VIS-NIR

spectrophotometer (Shimadzu, Kyoto, Japan).

Stability test of CCM-SPIO

The stability of CCM-SPIO was tested with the UV-VIS-NIR spectrophotometer by monitoring the change in optical absorption spectra. To this end, 100 μg mL^{-1} CCM-SPIO was dissolved in 1 mL PBS/FBS/DMEM and stored at room temperature. Optical absorption spectra were recorded every 12 h for 3 days.

Cell culture and animal model

GL261, LO2, b.End.3, and HUVEC cells were cultured separately in DMEM supplemented with 10% (v/v) FBS and 1% penicillin-streptomycin solution under a 5% CO_2 atmosphere and at 37°C. For animal models, male BALB/c nude mice and male C57/6N mice were purchased from Charles River (Beijing, China). Male BALB/c nude mice were used for MPI, MRI, and fluorescence imaging, and male C57/6N mice were used for *in vivo* antitumor tests. To create glioma model, Luciferase-tagged GL261 cells in a volume of 5 μL were injected into the right caudate nucleus of the BALB/c nude mice by a stereotaxic device. For C57/6N mice, 2×10^5 GL261 cells in about 50 μL PBS were subcutaneously injected into the hind leg positions of the mice to set up the tumor model. All animal procedures were carried out according to the guidelines approved by the Animal Ethics Committee of the Hong Kong Polytechnic University. The approval number for animal experiments is A0043201.

***In vitro* BBB model assay**

b.End.3 cells (1.0×10^5 cells/well) were seeded in the 12-well transwell plate with a membrane with a mean pore size of 0.4 μm to simulate the BBB environment. The transendothelial electrical resistance (TEER) values were recorded by the Millicell ERS-2 Epithelial Volt-Ohm Meter volt-ohmmeter (Millipore, USA) to assess the cell monolayer integrity during cell culture [1]. When the TEER value reaches 200 Ω cm^2 or above, it can be considered as an *in vitro* BBB model. Next, the fresh DMEM with uncoated SPIO and CCM-SPIO was replaced in the cells, respectively. Afterward, the medium in both apical and basolateral chambers was collected for ICP assays after another 6-hour incubation.

In vitro targeting studies

The cells were co-incubated with uncoated SPIO and CCM-SPIO for biological scanning electron microscopy. Following the incubation, DMEM was poured off from the dish without rinsing. The cells were immediately covered with electron microscope fixative and collected into a centrifuge tube by gently scraping them off the dish. After further addition of electron microscope fixative, the cells were fixed for 2 hours at room temperature and then transferred to a 4 °C refrigerator to be ready for subsequent experiments.

Biodistribution analysis

Pilot biodistribution images were taken using the In Vivo Imaging System (IVIS® SpectrumCT; PerkinElmer, Waltham, MA, USA) before and 1, 3, 6, 8, 10, 12, and 24 hours after injection of 200 μL CCM-SPIO-ICG (1 mg mL^{-1}) into the glioma-bearing mice to monitor the biodistribution of the nanoprobe *in vivo*.

Cytotoxicity assay

The cytotoxicity of the nanoprobes was tested on GL261 and LO2 cells. Cells were seeded at a density of 1×10^4/well in 96-well cell culture plates and incubated at 37°C under a 5% CO_2 atmosphere for 24 hours. Then, cells were treated with various concentrations (0, 5, 10, 20, 30, and 40 μg mL^{-1}) of CCM-SPIO (100 μL/well) for 24 hours. Finally, 100 μL/well of CCK-8/culture medium (10 μL/100 μL) was supplemented in each well and the plates were incubated for an additional 1 hr under the same conditions. A Synergy HT microplate reader (BioTek, Winooski, VT, USA) was used to measure the absorbance of each well at 450 nm (OD 450). The following formula was used to calculate the cell viability: Cell Viability (%) = [(As-Ab)/(Ac- Ab)] × 100%, where As, Ac, and Ab represent the OD 450 of the treatment group, control group, and blank, respectively.

Photothermal performance assay

Continuous 785-nm NIR laser illumination with a spot size of 5 mm was used to test the photothermal effect. The power density was 0.5 or 0.8 W cm^{-2}. Before irradiation, the samples were dissolved in deionized water to achieve Fe concentrations of 0, 5, 10, 20, 30, and 40 μg mL^{-1}, respectively. Then, 100 μL of each sample was used for photothermal measurements. The temperature variations of all samples were recorded using a FLUKE Ti25 infrared thermal imaging camera (Everett, WA, USA) at 30-second intervals, with an accuracy of 0.1°C. For optical stability detection, 20 and 40 μg mL^{-1} Fe concentrations in CCM-SPIO solutions were analyzed at 5-min intervals by fits and starts using the same laser conditions stated above. All experiments were conducted in triplicate.

***In vitro* PTT assay**

We used calcein-AM and propidium iodide (PI) staining to visually test the photothermal effect of CCM-SPIO. GL261 cells were cultured in 6-well plates at 37°C under a 5% CO_2 atmosphere for 24 hours. The original medium was washed off, followed by adding a medium containing 10 μg/mL or 20 μg mL^{-1} Fe in CCM-SPIO, whereas the medium without nanoprobes was used as a control. After 4 hours of continuous incubation, the cells were irradiated with a 785-nm laser (0.8 W cm^{-2}) for 5 mins in an already outlined area. Afterward, the cells were washed gently twice with 1× Assay buffer, followed by the addition of Calcein-AM and PI were added. The cells were incubated for another 15 mins and visualized by an inverted Leica M205 FA fluorescence microscope (Leica, Jena, Germany).

For quantitative analysis, GL261 cells were seeded at a density of 1×10^4/well in a 96-well plate at 37°C under a 5% CO_2 atmosphere for 24 hours. Then, the cells were randomly divided into four groups: control group, CCM-SPIO group, laser group, and CCM-SPIO with laser group. In the control group, the cells were replaced with a new routine culture medium as stated before. In the CCM-SPIO group and CCM-SPIO with laser group, the cells were treated with various concentrations (5, 10, 20, 30, and 40 μg mL^{-1} of iron) of CCM-SPIO for 4 hours. Subsequently, cells in the CCM-SPIO with laser group were washed three times with PBS and then exposed to 785-nm laser illumination at 0.8 W cm^{-2} for 3 mins. The same laser treatment procedure was also applied to the laser group but in the absence of CCM-SPIO. After that, CCK-8 was used as discussed earlier.

Statistical analysis

GraphPad Prism 8 software (GraphPad Software, San Diego, CA, USA) was used for statistical analysis. A t-test was performed to determine the differences among different groups. *p < 0.05, **p < 0.01, ***p < 0.001, and ***p < 0.0001 were considered statistically significant, whereas n.s. indicated no significant difference between the two selected groups.

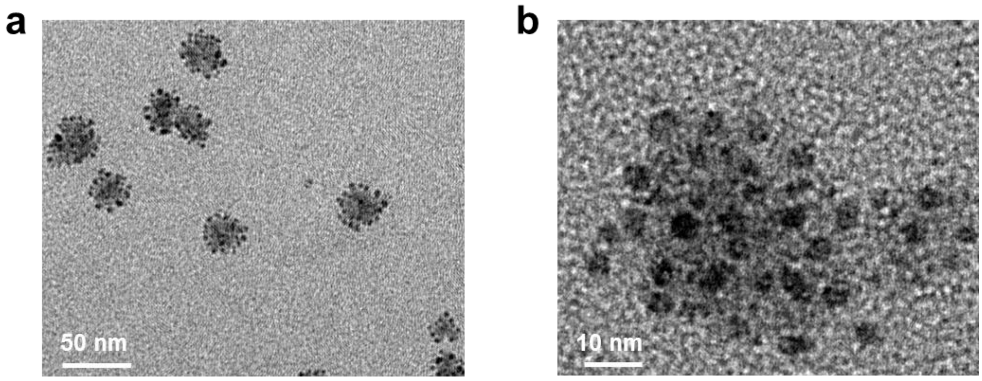

Figure S1. TEM images of SPIO

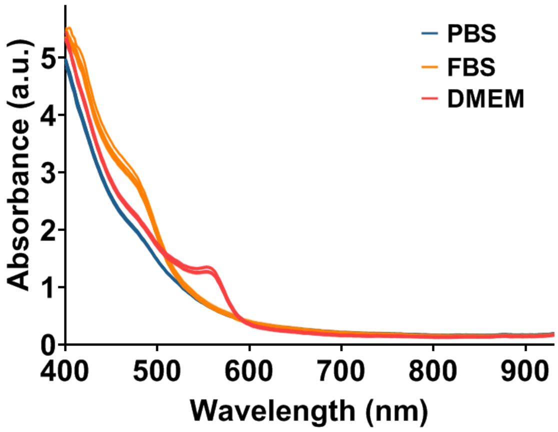

Figure S2: UV–Vis-NIR spectra of CCM-SPIO dissolved in the PBS/FBS/DMEM stored under room temperature for 3 days of testing. Each panel contained 6 sets of data at different time points.

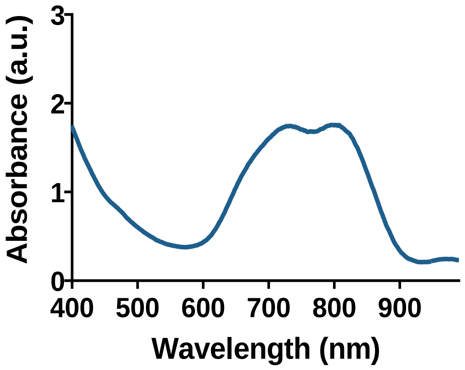

Figure S3. UV–Vis-NIR spectra of ICG-marked CCM-SPIO suspensions

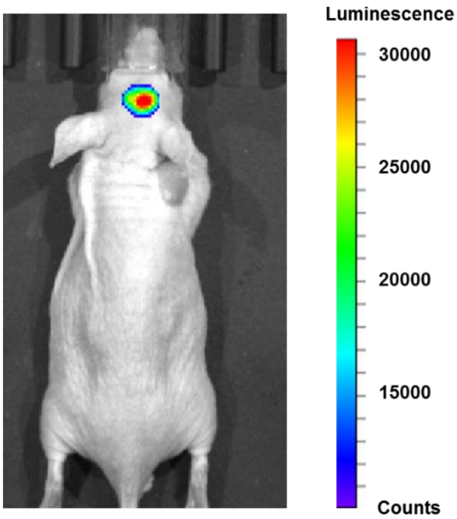

Figure S4. Auto-fluorescent images of mice orthotopic glioblastoma

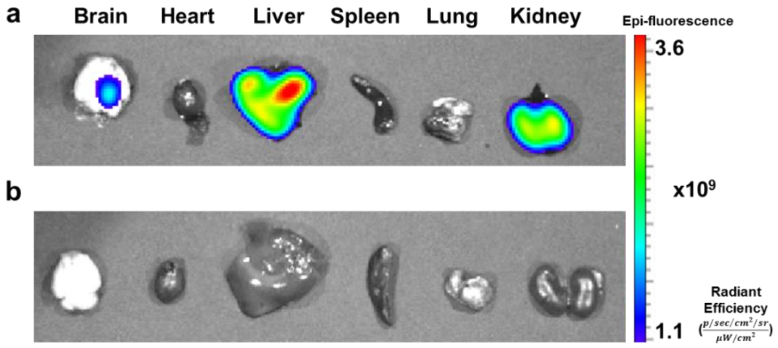

Figure S5. *Ex vivo* fluorescent imaging of mouse organs at 8 h (a) and 24 h (b) after ICG-loaded CCM-SPIO injection.

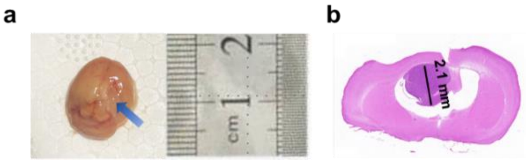

Figure S6. *Ex vivo* mouse brain tissue and the H&E slice of the maximum cross-section.

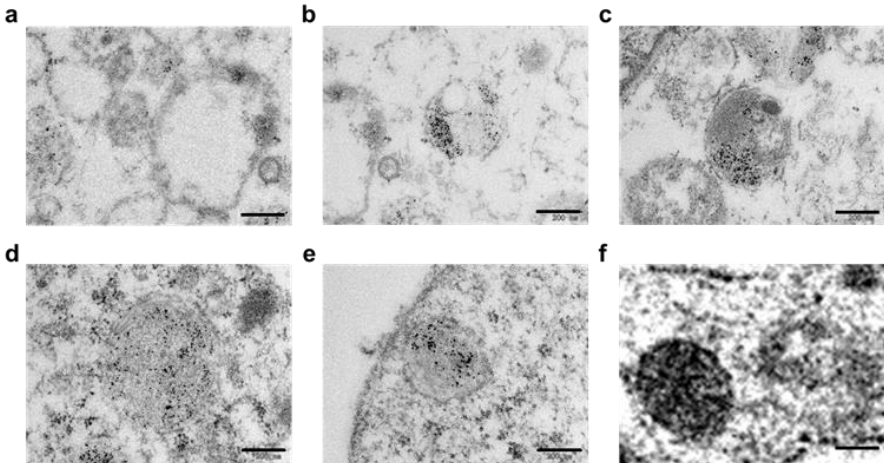

Figure S7. The cellular degradation of the CCM-SPIO at different stages was observed by TEM. a) Early endosome, b) late endosome, c-e) endo-lysosome, f) CCM-SPIO in the lysosome. Scale bar = 200 nm.

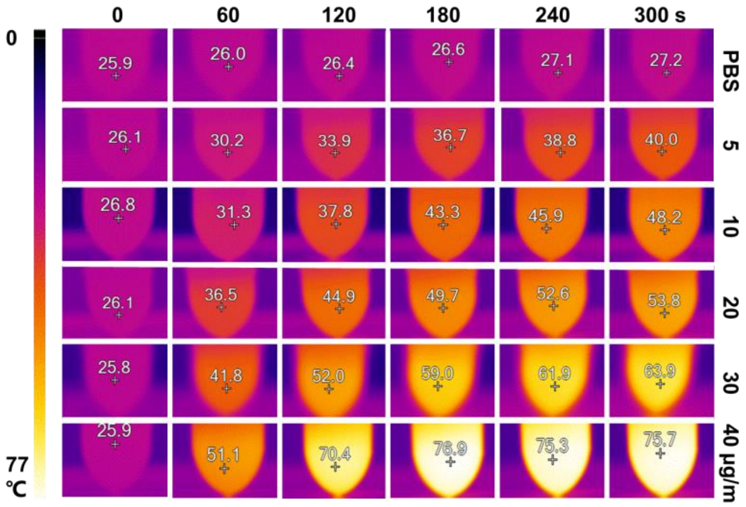

Figure S8. Temperature changes of various concentrations of the CCM-SPIO irradiated by 785 nm 0.6 W cm^{-2}. Images are captured by an infrared thermal camera.

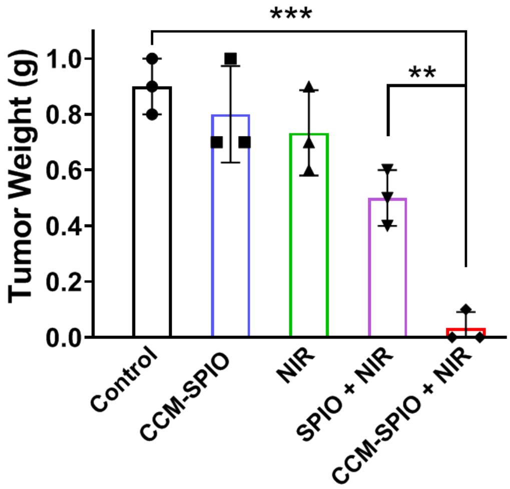

Figure S9. Quantitative analysis of the tumor weight from each group of mice.

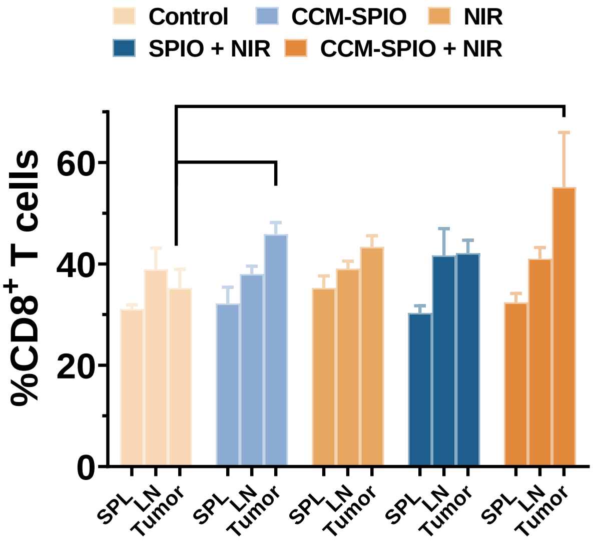

Figure S10. Percentage of CD3+CD8+ in T cells infiltrated in the treated tumors in each group.

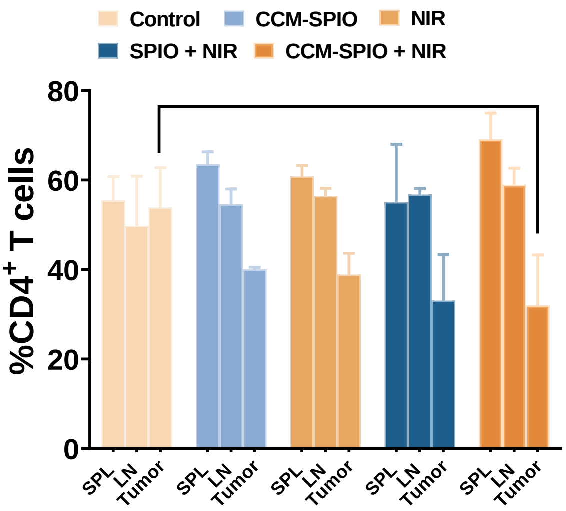

Figure S11. Percentage of CD3+CD4+ in T cells infiltrated in the treated tumors in each group.

Reference

[1] J. Xue, Z. Zhao, L. Zhang, L. Xue, S. Shen, Y. Wen, Z. Wei, L. Wang, L. Kong, H. Sun, Q. Ping, R. Mo, C. Zhang, *Nat Nanotechnol* **2017**, 12, 692.
